# A Rat α-Fetoprotein Binding Activity Prediction Model to Facilitate Assessment of the Endocrine Disruption Potential of Environmental Chemicals

**DOI:** 10.3390/ijerph13040372

**Published:** 2016-03-25

**Authors:** Huixiao Hong, Jie Shen, Hui Wen Ng, Sugunadevi Sakkiah, Hao Ye, Weigong Ge, Ping Gong, Wenming Xiao, Weida Tong

**Affiliations:** 1 Division of Bioinformatics and Biostatistics, National Center for Toxicological Research, U.S. Food and Drug Administration, Jefferson, AR 72079, USA; jieeshen@gmail.com (J.S.); Huiwen.Ng@fda.hhs.gov (H.W.N.); Suguna.Sakkiah@fda.hhs.gov (S.S.); haoye.ecust@gmail.com (H.Y.); weigong.ge@fda.hhs.gov (W.G.); Wenming.Xiao@fda.hhs.gov (W.X.); Weida.Tong@fda.hhs.gov (W.T.); 2 Environmental Laboratory, U.S. Army Engineer Research and Development Center, 3909 Halls Ferry Road, Vicksburg, MS 39180, USA; Ping.Gong@usace.army.mil

**Keywords:** model, prediction, α-fetoprotein, endocrine, disruption, binding, assessment

## Abstract

Endocrine disruptors such as polychlorinated biphenyls (PCBs), diethylstilbestrol (DES) and dichlorodiphenyltrichloroethane (DDT) are agents that interfere with the endocrine system and cause adverse health effects. Huge public health concern about endocrine disruptors has arisen. One of the mechanisms of endocrine disruption is through binding of endocrine disruptors with the hormone receptors in the target cells. Entrance of endocrine disruptors into target cells is the precondition of endocrine disruption. The binding capability of a chemical with proteins in the blood affects its entrance into the target cells and, thus, is very informative for the assessment of potential endocrine disruption of chemicals. α-fetoprotein is one of the major serum proteins that binds to a variety of chemicals such as estrogens. To better facilitate assessment of endocrine disruption of environmental chemicals, we developed a model for α-fetoprotein binding activity prediction using the novel pattern recognition method (Decision Forest) and the molecular descriptors calculated from two-dimensional structures by Mold^2^ software. The predictive capability of the model has been evaluated through internal validation using 125 training chemicals (average balanced accuracy of 69%) and external validations using 22 chemicals (balanced accuracy of 71%). Prediction confidence analysis revealed the model performed much better at high prediction confidence. Our results indicate that the model is useful (when predictions are in high confidence) in endocrine disruption risk assessment of environmental chemicals though improvement by increasing number of training chemicals is needed.

## 1. Introduction

Endocrine disruptors (EDs) are exogenous compounds that affect the endocrine system of humans and other vertebrates. Endocrine activity of environmental or foreign chemicals has the potential to cause numerous adverse outcomes, including disrupting the physiologic function of endogenous hormones and altering homeostasis. The known EDs include polychlorinated biphenyls (PCBs), the synthetic estrogen diethylstilbestrol (DES), dichlorodiphenyltrichloroethane (DDT) and other pesticides. For example, DES was approved the Food and Drug Administration (FDA) for treatment of menopausal symptoms, gonorrheal vaginitis, atrophic vaginitis, postpartum lactation suppression, and prostate cancer [1,2]. DES was shown to disrupt the endocrine system causing vaginal tumors in girls and women and other adverse medical complications [3] and thus was withdrawn from the market by the FDA. Concern about EDs has invigorated intense discussion and debate over the past two decades in the scientific community [4,5] and promoted the legislation for regulation of environmental chemicals mandated by the Environmental Protection Agency (EPA) and development of the Endocrine Disruptor Screening Program (EDSP) to screen potential EDs in the environment [6].

EDs can disrupt the endocrine system through different mechanisms [7,8,9,10,11,12]. One of the well-known mechanisms is mediated by the hormone receptors such estrogen receptor (ER) and androgen receptor (AR), in which EDs exhibit their estrogenic and androgenic effects through binding to the ER and AR in the target cells [13,14,15,16]. Therefore, a huge amount of estrogenic and androgenic activity data of structurally diverse chemicals have been generated and organized in sophisticated databases such as the FDA’s Endocrine Disruptors Knowledge Base (EDKB) [17] and Estrogenic Activity Database (EADB) [18]. These databases have been used for the development of a diverse set of quantitative structure-activity relationship (QSAR) models for predicting estrogenic and androgenic activity and to assist evaluation of endocrine disruption potential of environmental chemicals [19,20,21,22,23,24,25,26,27,28,29].

ED binding to hormone receptors in target cells is the key mechanism to display endocrine disruption. However, the affinity of binding to ER is not the sole criterion to determine EDs’ potential to disrupt the endocrine system. For example, EDs cannot bind to ER or AR in the target cells if they cannot pass the cell membrane. Therefore, *in vitro* ER and AR binding data of chemicals may not reflect well their *in vivo* endocrine activity, even for chemicals with high *in vitro* binding affinity. To accurately estimate the endocrine disruption potential of environmental chemicals, it is necessary to have both their binding activities to hormone receptors and to competing serum proteins such as alpha-fetoprotein (AFP) [30,31] and human sex hormone-binding globulin (SHBG) [32].

There are different transporter proteins in serum, including albumin, globulin, fibrinogen, and others. The transporter proteins can transport hormones, vitamins and other chemicals within and between cells and organs. SHBG is one of the major transporter proteins that bind to hormones and other chemicals in human serum [33]. AFP is a major transport protein in rat and was first discovered approximately 60 years ago [34]. It is a serum biomarker of Down’s syndrome and neural tube defects in the clinical practice and alters the growth of fetal and cancer cells [35,36]. Entrance of AFP into cells through receptor-mediated endocytosis was observed in fetal cells of different species including rat [37], mouse [38], human [39] chicken [40] and baboon [41]. Elevated AFP level was observed in maternal circulation through transplacental passage from the fetal circulation and amniotic fluid by the placental or allantois [42,43,44,45]. This protein competes with ER to bind estrogens in the blood and thus inhibits EDs access to the target cells [46,47]. It has been found that diverse chemicals bind AFP [30,48,49,50,51,52].

A huge amount of *in vitro* binding assays data have been generated for the targets such as ER and AR involved in the endocrine system. However, available *in vivo* bioactivity data related to endocrine disruption potential are relatively less than the *in vitro* data. Moreover, most of the *in vivo* data are obtained using rats uterotrophic assays [17,18,53]. To better assess endocrine disruption potential of environmental chemicals, we measured rat AFP binding affinity for 125 chemicals with diverse structures using a competitive binding assay according to the methods published in our previous study [30]. Our rat AFP binding data represent the largest such data set to date. Compared with the experimental data on the hormone receptors such as ER and AR, there are fewer chemicals with experimental AFP binding data, hindering the risk assessment of environmental chemicals in terms of endocrine disruption potential. Therefore, for an enhanced risk assessment it was necessary to obtain AFP binding data for those environmental chemicals lacking AFP binding data. To this end, we developed an *in silico* model for prediction of AFP binding activity of environmental chemicals using our previously reported data [30]. The performance of the model was internally evaluated through cross validations and permutation tests. It was also validated externally using the AFP binding activity data curated from the literature. We demonstrated that the model has suitable predictive power and is expected to better assist endocrine disruption assessment of environmental chemicals.

## 2. Materials and Methods

### 2.1. Study Design

**Figure 1:**
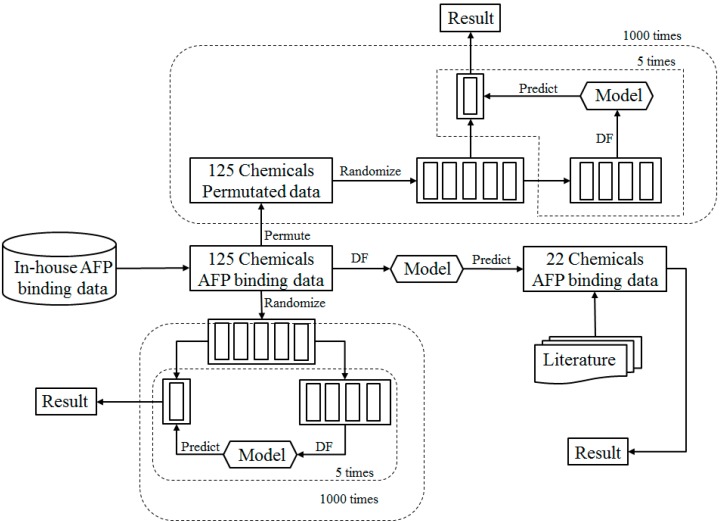
Overview of the study design.

The study design is depicted in Figure 1 and the detail explanation for each step is described in the following sections. Briefly, the 125 chemicals and their rat AFP finding activity (53 binders and 72 non-binders) from our previous study [30] were used as the training data set. First, 5-fold cross validations were conducted to evaluate the performance of Decision Forest (DF) model as illustrated in the bottom left part of Figure 1. More specifically, the training data set were randomly divided into five equal portions of chemicals. Four portions were used for training the DF model and the remaining portion was used for testing the DF model. The process was repeated five times so that each of the five portions was used as test data set to challenge the models that were constructed from the other four portions. The prediction results from the five DF models were averaged to estimate the models’ performance. To reach a statistically robust estimation of the DF models’ performance, the 5-fold cross validation process was iterated 1000 times. The resultant data from the 1000 iterations of 5-fold cross validation were used for prediction confidence analysis and identification of informative molecular descriptors that are important for AFP binding. Then, permutation tests were conducted to affirm that the prediction accuracy observed in the 5-fold cross validations was not achieved by chance, as illustrated in the top part of Figure 1. In brief, the binding activity data (binder or non-binder) of the 125 chemicals in the training data set were permutated first and a 5-fold cross validation was carried on the resultant permutated data set. The permutation test was repeated 1000 times to make sure that the permutation tests result is statistically robust. Finally, the whole training data set was used to train a DF model that was validated using an external data set. The external validation data set was curated from the literature [48,49,50].

### 2.2. Data Sets

The 125 structurally diverse chemicals with rat AFP competitive binding assay results published earlier [30] were used as the training data set. Of the 125 chemicals, 53 chemicals displayed binding affinities to rat AFP. The IC_50_ values of the 53 chemicals are in the range of 0.0065 to 590 nM. All of the 53 chemicals were defined as AFP binders in this study. The rest 72 chemicals did not show binding affinity to rat AFP and were determined to be AFP non-binders. In this study, binders were represented by “1” and non-binders by “0” in the model constructions and predictions. The two-dimensional (2D) structures of the 125 chemicals were generated according to our previous study using Marvin Sketch (http://www.chemaxon.com/) and saved in a single 2D SDF (structure-data file) format file [30].

For validation of AFP binding activity prediction model, we curated an external data set through literature search for AFP binding activity. First, the chemicals with AFP binding activity data were collected from the literature. After removing the chemicals that were presented in the training data set, 22 chemicals with known AFP binding activity data from other studies [48,49,50] were used as the external validation set. The structures of the 22 chemicals were drawn according to the literature using Marvin Sketch and saved in a single 2D SDF format file.

### 2.3. Molecular Descriptors

QSAR models are developed based on different types of molecular descriptors. The molecular descriptors of the chemicals in both training and external validation data sets were generated using Mold^2^ [54,55]. Mold^2^ is a free software which calculates molecular descriptors from 2D chemical structures. This software is very fast because it adopts the extremely rapid algorithm for cyclic structure recognition [56] and uses the efficient chemical structure representation system [57,58] that has shown high efficiency in the system for chemicals structure elucidation based on infrared [59] and nuclear magnetic resonance (NMR) spectra [60,61,62]. Mold^2^ has been demonstrated to be reliable for developing QSAR models [63,64]. In brief, 777 Mold^2^ descriptors were first calculated for each of the chemicals in the training and external validation data sets. Then, the descriptors were cleaned up by removing those with constant values across all the chemicals in the data sets. Finally, the remaining 512 Mold^2^ descriptors were scaled to the values between 0 and 1.

### 2.4. Prediction Model

Prediction models can be developed using different QSAR methods such as pharmacophore modeling [65,66,67,68], molecular docking [69,70] and machine learning methods [71,72,73]. In this study, the prediction models were built using the Mold^2^ descriptors and the pattern recognition algorithm DF that was developed previously by our group [74,75]. DF is a free software for public use [76] that employs a consensus modeling technique by combining multiple decision tree models. It uses a unique procedure to construct different decision tree models to ensure heterogeneous models when combined. Besides, variable selection process is wrapped in the model construction process, which simplifies the model development. In addition to QSAR, the DF algorithm were applied for the development of predictive models based on the genomics data [77,78] and proteomics data [79]. The DF models in this study were constructed using the following algorithmic parameters: the number of trees is set to 5; the minimum size of node to be split is 10; the maximum levels to be pruned to is 3; and the method for node splitting is Gini’s diversity index. The tree building and pruning processes were guided by achieving the minimum number of misclassified compounds.

### 2.5. Cross Validations

To assess the performance of the DF model, 5-fold cross validations were conducted as illustrated in Figure 1. In one 5-fold cross validation, the 125 chemicals of the training data set were randomly divided into five equal portions. Four of the five portions were used to construct a DF model, which was then used to predict AFP binding activity for the chemicals in the remaining one portion. This process was repeated sequentially so that each of the five portions was left out once and only once as the testing set. The prediction results from the five testing sets were then averaged as an estimate of the DF model performance using accuracy, sensitivity, specificity, Matthews correlation coefficient (MCC) and balanced accuracy. These performance metrics were calculated using Equations (1)–(5) through comparison of the predictions with the actual AFP binding activity data:

(1) $$Accuracy=\frac{TP+TN}{TP+TN+FP+FN}$$

(2) $$Sensitivity=\frac{TP}{TP+FN}$$

(3) $$Specificity=\frac{TN}{TN+FP}$$

(4) $$MCC=\frac{TP\times TN-FP\times FN}{\sqrt{\left(TP+FP)(TP+FN)(TN+FP)(TN+FN\right)}}$$

(5) $$Balanced\cdot Accuracy=\frac{TP(TN+FP)+TN(TP+FN)}{2(TP+FN)(TN+FP)}$$

In Equations (1)–(5), true positive (*TP*) is the number of AFP binders that were predicted as binders by the DF models, true negative (*TN*) is the number of AFP non-binders that were predicted as non-binders, false negative (*FN*) is the number of AFP binders that were predicted as non-binders, and false positive (*FP*) is the number of AFP non-binders that were predicted as binders.

### 2.6. Permutation Tests

Permutation analysis is a common approach to determine whether the model performance estimated from cross validations is due to chance correlations. As shown in Figure 1, in one permutation test, the qualitative AFP binding activity data (1 for binder and 0 for non-binder) of the 125 chemicals in the training data set were randomly shuffled while the Mold^2^ descriptors values (the independent variables) remained unchanged to generate a permutated data set. A 5-fold cross validation described above was then conducted on the permutated data set and the cross validation results were compared with the results from the cross validations on the real training data set. The permutation test was repeated 1000 times by using different randomly shuffled AFP binding activity data to reach a statistically significant robust comparison with the 1000 times of 5-fold cross validations using the real training data set.

### 2.7. Prediction Confidence Analysis

In the cross validations, the AFP binding activity prediction from a DF model for a chemical is a continuous value, *p*, that is used to forecast the qualitative AFP binding activity of the chemical as AFP binder (*p* ≥ 0.5) or non-binder (*p* < 0.5). This value indicates the likelihood of the chemical to be a AFP binder or AFP non-binder and represents the confidence for the prediction. A good prediction model is expected not only to show accuracy but also to predict most unknown chemicals with high confidence level. Furthermore, the predictions with a higher prediction confidence level should be more accurate than the predictions at a lower prediction confidence level. We analyzed the relationship between the prediction confidence and the corresponding prediction accuracy of the DF models in the 1000 iterations of 5-fold cross validations using the training data set. The prediction confidence was calculated for each of the predictions from the 1000 times of 5-fold cross validations using Equation (6).

(6) $$Confidence=\frac{\left|p-0.5\right|}{0.5}$$

The calculated prediction confidence is a value between 0 and 1. The larger the value, the more reliable is the prediction. The predictions of the 5-fold cross validations were placed into 20 groups with even confidence bins. For each of the 20 groups of predictions, prediction performance metrics such as sensitivity, specificity and accuracy were calculated by comparing the predictions with the actual AFP binding activity data. At last, the performance of the DF models at difference confidence levels was analyzed.

### 2.8. Informative Molecular Descriptors Identification

Generally, QSAR models are built for one or both of the purpose of prediction and/or to gain mechanistic understanding of biochemical phenomena [80]. Mechanistic understanding is derived from the ability to interpret the physicochemical meaning of molecular descriptors used in QSAR models. To better understand the chemical aspects that play important roles in the binding interactions with AFP, the molecular descriptors used in the DF models were examined to identify the Mold^2^ descriptors that are informative to the DF models. First, the frequency values of the Mold^2^ descriptors that were used in the DF models in the 1000 permutation tests were calculated to establish a statistical background. The DF models were constructed from the random data sets obtained by permutation and, thus, the top 5% frequency can be used as the frequency criterion to identify the informative descriptors with a 5% probability for the descriptors being selected due to the random noises, that is at a *p*-value = 0.05. Then, the frequency values of the Mold^2^ descriptors that were used in the DF models in the 5-fold cross validations were computed and compared with the frequency of 0.05 (*p*-value) that was determined from the permutation tests. The Mold^2^ descriptors that had higher frequency values than the frequency of 0.05 (*p*-value) were identified as the informative descriptors for AFP binding activity prediction.

### 2.9. External Validation

QSAR models usually perform better on the dataset that was used to construct the models in cross validations than on new data. Validation using external data sets is important and necessary to assess the performance of a predictive model. In this study, 22 chemicals with known AFP binding activity data from the literature were assembled for external validation. The predictive DF model was built on the entire training data set of 125 chemicals and then used to predict the AFP binding activity of these 22 chemicals in the external validation set.

## 3. Results

### 3.1. Cross Validations

**Figure 2:**
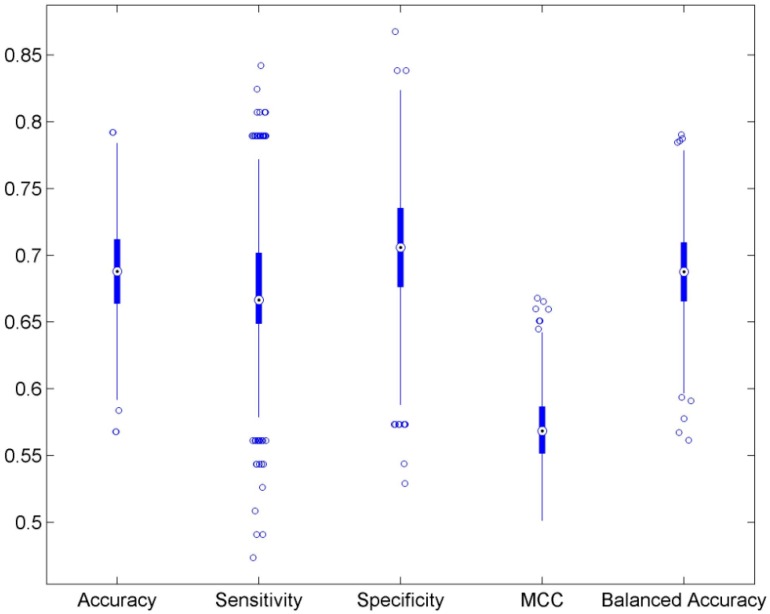
Boxplots for the predictions from the DF models in the 5-fold cross validations. Performance were measured by metrics as indicated on the *x*-axis.

We conducted 1000 5-fold cross validation cycles using the training data set as shown in Figure 1. The prediction results from the DF models were compared with the actual AFP binding activity data to calculate the metrics for evaluation of the performance of the models. The 5-fold cross validation results were plotted in the boxplots of Figure 2 and are summarized in Table 1. The average values of accuracy, sensitivity, specificity, MCC and balance accuracy are 68.9%, 67.5%, 70.0%, 57.0% and 68.8% respectively. All performance metrics indicate a moderate prediction power of the DF models. The small standard deviation values obtained demonstrated that the DF models are statistically robust.

### 3.2. Permutation Tests

**Figure 3:**
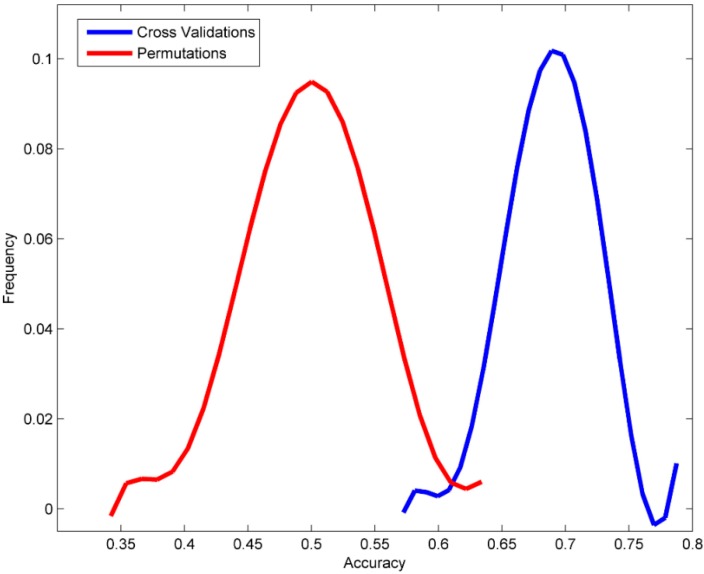
Distributions of the 1000 prediction accuracy values calculated from the DF models in permuation tests (red line) and yielded from the DF models in the cross validations (blue line).

Permutation tests were conducted to affirm that the prediction power observed for the DF models in the 5-fold cross validations was not due to chance correlation in the training data set. The prediction results from the DF models that were constructed using the 1000 permutated datasets and were plotted for the distribution of prediction accuracy values as the red line in Figure 3. For comparison, the distribution of the prediction accuracy values from the 1000 times of 5-fold cross validations is represented as the blue line in Figure 3. Obviously, the predictions from the cross validations were significantly more accurate than the predictions from the permutation tests, with a *p*-value < 0.0001. The same difference were observed for other metrics: the differences between the average values of the cross validations and the permutation tests were 19.0%, 24.7%, 14.2%, 7.3% and 19.5% in overall accuracy, sensitivity, specificity, MCC and balanced accuracy respectively. Therefore, the permutation tests demonstrated that the AFP binding activity predictions of the DF models in the cross validations were not obtained by probability success.

### 3.3. Prediction Confidence Analysis

We analyzed the prediction confidence using the 1000 times of 5-fold cross validations. The confidence levels of the predictions from the DF models in the 1000 iterations of 5-fold cross validations were calculated and used to place the predictions into 20 groups with even confidence bins. Correct and incorrect predictions were then counted for each of the 20 groups by comparison with the actual AFP binding activity data.

**Figure 4:**
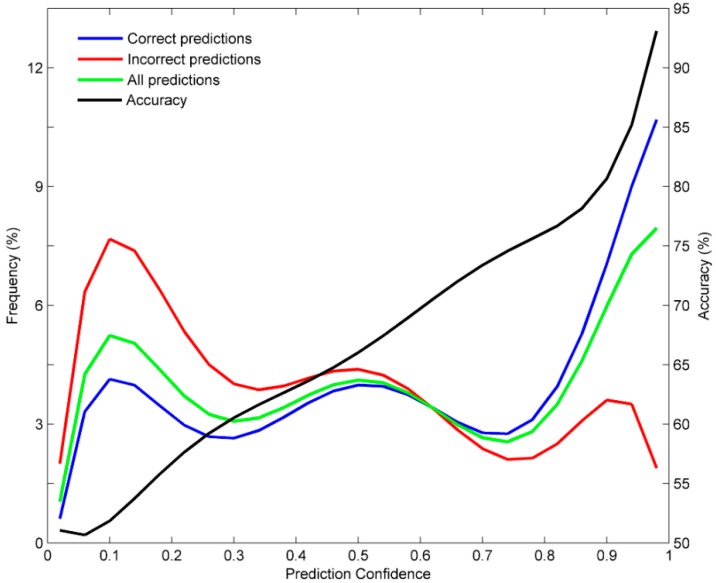
Predictions and accuracy at different confidence levels. The distributions of predictions were given by the left *y*-axis and the prediction accuracy is indicated by the right *y*-axis. Prediction confidence was given at the *x*-axis. Predictions are plotted in green line, correct predictions in blue line, incorrect predictions in red line, and prediction accuracy in black line.

Prediction accuracy was calculated for the predictions in each of the 20 groups. The numbers of predictions, correct predictions and incorrect predictions for the 20 groups were shown as blue, red and green distribution curves respectively in Figure 4.

The corresponding prediction accuracy values for the 20 groups were plotted as a black distribution line in Figure 4. As the confidence level increased, the correct predictions increased (blue line) while the incorrect predictions reduced (red line). More importantly, it was found that higher the prediction confidence, the more accurate are the predictions (black line). Moreover, most predictions from the DF models were at high confidence (green line). The prediction confidence analysis demonstrated that the DF models not only had a reasonable prediction power but also gave prediction confidence values that could be utilized to better assist evaluation AFP binding activity of chemicals.

### 3.4. Identification of Informative Descriptors

**Figure 5:**
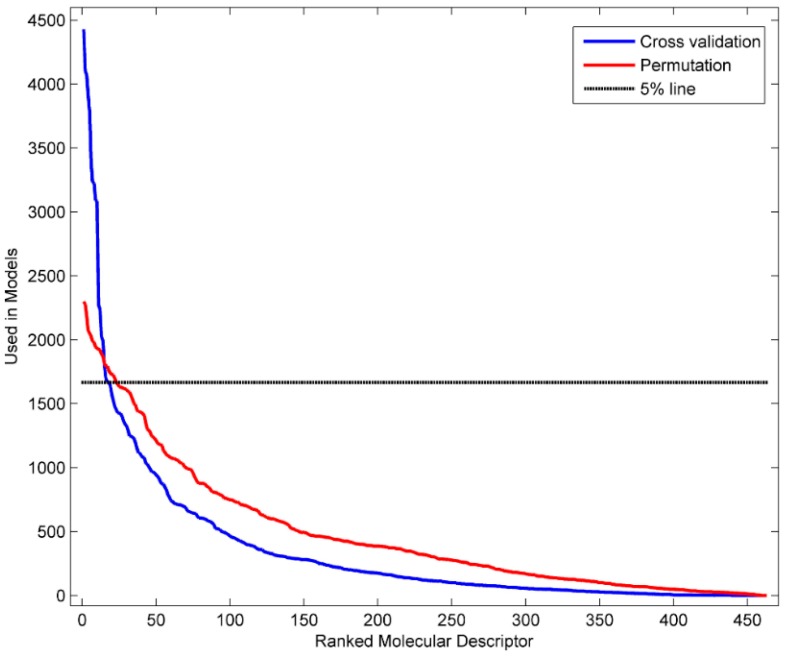
The distribution of descriptors used in the DF models.

The more frequently a descriptor is used in QSAR models, the more informative it is to the QSAR models. The informative molecular descriptors are important for interpretation of QSAR models. To identify the informative descriptors to the DF models in the 5-fold cross validations, we first extracted the Mold^2^ descriptors that were actually used in the models. Then, the frequency of each of the 512 Mold^2^ descriptors used by the 5000 DF models was calculated. The results were plotted as the solid blue line in Figure 5. Similarly, the frequency of each Mold^2^ descriptor used in the 5000 DF models in the permutation tests was calculated. The results were displayed as the solid red line in Figure 5. The top 5% descriptors in the permutation tests were separated by the dotted black line at a frequency of 1680 models in Figure 5. Therefore, the Mold^2^ descriptors that were used in more than 1680 DF models in the 5-fold cross validations should be informative to the DF models at the 5% significance level in a statistical view. Using this cut-off, 16 Mold^2^ descriptors that were used by more than 1680 DF models were identified as the informative descriptors. Table 2 lists these 16 Mold^2^ descriptors, the numbers of DF models, and the descriptor definitions.

The identified informative descriptors are the indices that are related to molecular shape, electronegativity and polarizability of the chemicals. Therefore, the molecular shape of a chemical and its hydrophilic interactions with the ligand binding pocket of AFP are the key structural features that determines if a chemical can bind to AFP. This finding is consistent with our previous structural analysis of AFP ligand binding pocket [32].

### 3.5. Prediction Model and External Validation

**Figure 6:**
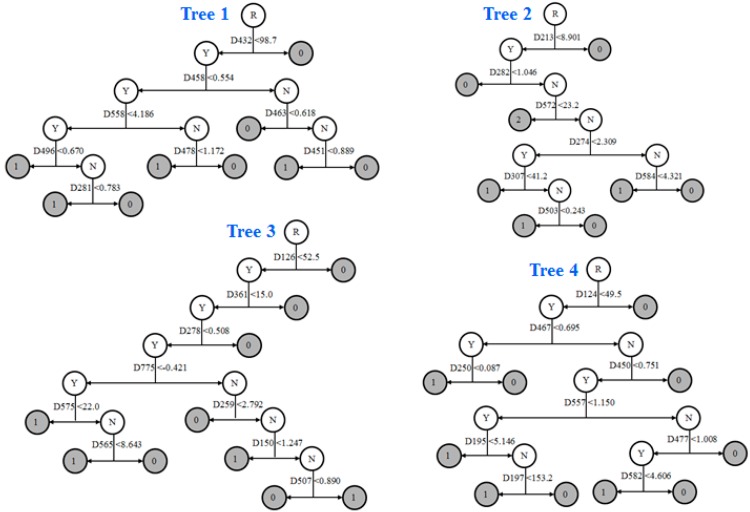

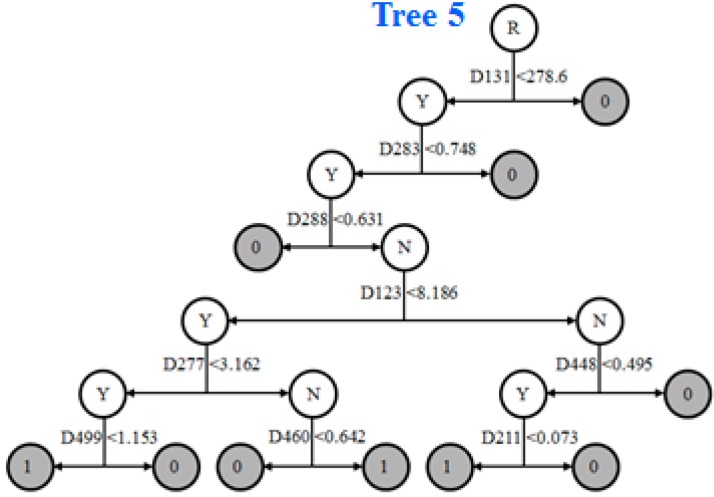
Decision trees of the AFP binding activity prediction DF model. The descriptors and their criteria that were used to split the intermediate nodes are given under the nodes. The left nodes are the sets of chemicals that meet the criteria for splitting their parent nodes; the right nodes represent the sets of chemicals that do not meet the criteria. The root node (whole training data set) and the intermediate nodes are presented in empty/white circles. Letter Y in a circle indicates the chemicals in the node meet the splitting criterion, whereas the letter N means the chemicals do not meet the splitting criterion. The terminal nodes are the leaves of the trees where the AFP binding activity predictions were determined and are shown in grey circles. Number 1 in a circle indicates that the chemicals in the node are predicted as AFP binders while number 0 marks the node where chemicals are predicted as AFP non-binders.

The AFP binding activity prediction DF model was constructed using the 125 chemicals of the training data set. The DF model consisted of five decision trees that are illustrated in Figure 6. The trees had eight to ten terminal nodes. The DF model was used to predict AFP binding activity for the 22 chemicals from the external data set. The 22 chemicals, including their names used in the literature, experimental AFP binding data, DF model prediction results and the references are given in Table 3.

The predictive performance of the DF model on the external validation set was measured using five different metrics: overall prediction accuracy, sensitivity, specificity, MCC and balanced accuracy. The calculated performance metrics for the external validation are listed Table 1. Slightly lower performance was observed for the external validation compared to the performance of the 5-fold cross validations.

## 4. Discussion

AFP is a protein in the plasma that binds to estrogens with high affinity. It can sequester EDs in the plasma and thereby reduces the concentration of EDs that can enter into the target cells. Thus, AFP can protect EDs in maternal circulation. Hence, AFP binding activity of chemicals is important information for assessment of endocrine disruption potential. If a chemical does not bind to AFP but binds to hormone receptors such as AR and ER, it can bypass AFP protection and has the potential to disrupt the endocrine system. In contrast, if a chemical binds to AFP, AFP could protect against endocrine disruption even if it has the potential to bind AR or ER. However, a very limited number of chemicals have been experimentally assayed for their AFP binding activity. Thus, we previously measured AFP binding activity for 125 structurally diverse chemicals using the competitive assay developed from rat amniotic fluid [30]. The number of chemicals with AFP binding activity data is still much smaller than the chemicals having ER and AR binding activity, hampering comprehensive assessment of endocrine disruption potential for environmental chemicals. Therefore, in this study, we developed and extensively validated AFP binding activity prediction models using the data published in the literature including our in-house data set. Our model showed a reasonable predictive power and robustness and could be expected to help assess endocrine disruption potential of environmental chemicals.

The DF prediction model was constructed using rat AFP binding data. It could be used for prediction of rat AFP binding activity for the environmental chemicals that have no experimental data. However, the limitation of current model should be noticed when applying the model in applications of human risk assessment of environmental chemicals because the human AFP is not completely homologous to the rat AFP.

Prediction confidence analysis showed that the DF models predicted AFP binding activity very accurately for some chemicals but not so well for other chemicals. The higher the prediction confidence, more likely the prediction is accurate as demonstrated in Figure 4. Therefore, we suggest that the AFP binding activity prediction (binder or non-binder) should be combined with the prediction confidence to better apply the DF model in assessment of endocrine disruption potential of environmental chemicals.

Though AFP was identified long time ago and has been extensively studied, no three-dimensional structure (3D) of AFP or complexes of AFP bound to ligands has been determined by X-ray crystallization. The structural features of this protein, especially in its ligand binding domain, were understood based only on the experimental binding activity data. Therefore, a homology model of rat AFP was constructed and the ligand binding interactions of this protein were elucidated using molecular docking and molecular dynamics simulations in our previous study [31]. The computationally constructed 3D structure of rat AFP and the *in silico* elucidated ligand binding interactions are expected to help the estimated AFP binding activity of environmental chemicals. Our previous study identified two different binding pockets in rat AFP, consistent with the two putative estrogen binding sites in AFP [81]. The ligand binding interactions of rat AFP contribute from residues Glu206, Glu209, Gly210, Leu213, Lys236, His260, Try306 and His310 in the first binding site and from residues Leu233, Gln239 and Glu312 in the second binding site [31]. Most of these amino acids have charged or have polar residues. Thus, hydrophilic and electrostatic interactions are important for a chemical to bind to AFP. Furthermore, the binding pockets were found to be different in size and shape. In this study, 16 Mold^2^ descriptors (Table 2) were identified as the informative descriptors to the DF prediction models. Therefore, these molecular descriptors represent the important structural features that are determinant to AFP binding activity of chemicals. The 16 Mold^2^ descriptors are the structural features of the chemicals interacting with AFP related to molecular shape, electronegativity, and polarizability of chemicals indicating molecular shape, hydrophilic and electrostatic interaction capability. These molecular characteristics are used to differentiate AFP binders from non-binders. The informative descriptors identified in this study confirmed the reliability of our previously constructed 3D structure of rat AFP and the elucidated ligand binding interactions.

Recently EPA considered utilization of high throughput screening assays and computational models in the endocrine disruptor screening program [82]. EPA led CERAPP project to develop QSAR models for prediction of estrogenic activity and the models were used for prioritize environmental chemicals for Tier-2 testing [83]. With binding data of transporter proteins obtained from experiments or *in silico* predictions, it is speculated that better priority setting the environmental chemicals for testing would be yielded.

The DF prediction models showed lower prediction accuracy than the DF model we previously developed for prediction of ER binding activity [20]. The less predictive power of the AFP binding activity prediction models may be partially due to the relatively small sample size. We expected more accurate DF prediction models would be constructed when AFP binding activity is experimentally measured for more chemicals that can be used as training samples. Another speculation on the cause of the relatively low prediction accuracy is the multiple binding sites in AFP. The 125 chemicals bind AFP in different interaction regions. The first ligand binding site in rat AFP lies in the region of amino acids 419–433 and the second ligand binding site consists of amino acids 450–464. The chemicals that displayed rat AFP binding activity in our previous study are structurally diverse [30]. The existence of two distinct ligand binding sites in AFP indicates that prediction of binding activity of a chemical depends on the AFP site where the chemical binds [84,85]. Therefore, we assume separate prediction models should be developed, each for one of the two ligand binding sites, to improve the performance of AFP binding activity prediction model. Our previous study demonstrated competitive modeling based on molecular docking may perform better than the DF modeling for AFP binding prediction. Lack of knowledge on the binding sites for chemicals and the limited number of experimental binding data available is a major impediment in the development of such separate prediction models. Our results indicated that simple predictive models such as the DF models in this study sometimes yield inaccurate predictions, especially when the system in modeling is not simple. Even though a moderate prediction power has been shown for the AFP binding activity prediction DF model, caution is warranted in application of the DF model in assessment of endocrine disruption potential of environment chemicals, especially when a prediction has a low prediction confidence. Nonetheless, the rat AFP binding activity predictions of high confidence from the DF models should be useful for assistance in estimation of rat AFP binding activity of environmental chemicals.

## 5. Conclusions

Using a set of structurally diverse chemicals whose rat AFP binding activity data were measured in our previous study, a DF model for prediction of the AFP binding activity was developed in this study. Internal cross validations and external validations were conducted to demonstrate the accuracy and robustness of the models. Our results showed a moderate prediction performance of the models. More importantly, the DF model provides prediction confidence that is very useful when applying the model in assessment of endocrine disruption potential of environment chemicals.

## Figures and Tables

**Table 1:** Summary of cross validations, permutation tests, and external validation.

Parameter	Cross Validations		Permutation Tests		External Validation
	Mean	STD	Mean	STD	
Accuracy	0.689	±0.034	0.498	±0.049	0.546
Sensitivity	0.675	±0.054	0.427	±0.067	0.412
Specificity	0.700	±0.046	0.558	±0.061	1.000
MCC	0.570	±0.026	0.497	±0.009	0.371
Balanced accuracy	0.688	±0.034	0.492	±0.050	0.706

STD: standard deviation.

**Table 2:** Informative descriptors identified from the cross validations.

ID	Models	Descriptor Definition
D282	4429	complementary information content (neighborhood symmetry of 2-order)
D281	4099	structural information content (neighborhood symmetry of 2-order)
D450	4075	Geary autocorrelation-lag 4/weighted by atomic masses
D432	3916	Broto-Moreau autocorrelation of a topological structure-lag 2/weighted by atomic Sanderson electronegativity
D458	3770	Geary autocorrelation-lag 4/weighted by atomic van der Waals volumes
D361	3391	ratio of multiple path counts to path counts
D213	3233	valence connectivity index chi-1
D467	3225	Geary autocorrelation-lag 5/weighted by atomic Sanderson electronegativity
D491	3091	Moran autocorrelation-lag 5/weighted by atomic van der Waals volumes
D259	3084	mean information content on the distance degree equality
D496	2272	Moran autocorrelation-lag 2/weighted by atomic Sanderson electronegativity
D478	2238	Geary autocorrelation-lag 8/weighted by atomic polarizabilities
D463	2024	Geary autocorrelation-lag 1/weighted by atomic Sanderson electronegativity
D246	1995	Maximum of the differences between vertex distance and unipolarity
D473	1799	Geary autocorrelation-lag 3/weighted by atomic polarizabilities
D595	1698	highest eigenvalue n. 8 of Burden matrix/weighted by atomic polarizabilities

**Table 3:** The experimental and predicted AFP binding activity of the external data set.

Chemical Name	Experiment	Prediction	Reference
17-α-Ethynylestradiol	1	1	[49]
11-β-Ethyloxyestradiol	1	0	[48]
11-β-Methoxyestradiol	1	1	[48]
Compound **7b**	1	0	[49]
16-α-Fluoroestradiol (FES)	1	1	[48]
Compound **8b**	1	0	[49]
Compound **8c**	1	1	[49]
Compound **3**	1	1	[48]
Compound **1**	1	0	[48]
Compound **2**	1	0	[48]
Compound **7c**	1	1	[49]
11-β-Ethyl-17-α-ethynylestradiol	1	0	[49]
11-β-Ethylestradiol	1	0	[49]
Compound **8a**	1	0	[49]
17-α-Ethynyl-11-β-Methoxyestradiol	1	0	[49]
Compound **7a**	1	0	[49]
4-Nonylphenoxyacetic acid (NP1EC)	1	1	[50]
4-*tert*-Butylphenol (BP)	0	0	[50]
Igepal	0	0	[50]
2,4’DDT	0	0	[50]
2,4’-DDE	0	0	[50]
Kepone	0	0	[50]

AFP binding data: 1 represents binder and 0 indicates non-binder.

## Abbreviations

The following abbreviations are used in this manuscript:

2D	Two-Dimensional
3D	Three-Dimensional
AFP	Alpha-Fetoprotein
AR	Androgen Receptor
EADB	Estrogenic Activity Database
ED	Endocrine Disruptor
EDKB	Endocrine Disruptors Knowledge Base
EDSP	Endocrine Disruptor Screening Program
EPA	Environmental Protection Agency
DF	Decision Forest
ER	Estrogen Receptor
FDA	Food and Drug Administration
FN	False Negative
FP	False Positive
MCC	Matthews Correlation Coefficient
NMR	Nuclear Magnetic Resonance
QSAR	Quantitative Structure-Activity Relationship
SDF	Structure-Data File
SHBG	Sex Hormone-Binding Globulin
STD	Standard Deviation
TN	True Negative
TP	True Positive
